# Decoding the Principles Governing Molecular Cage Precipitation of Aliphatic and Perfluoroalkyl Acids (PFAAs)

**DOI:** 10.1021/acsami.6c01610

**Published:** 2026-03-10

**Authors:** María Pérez-Ferreiro, Alejandro Criado, Jesús Mosquera

**Affiliations:** CICA—Centro Interdisciplinar de Química e Bioloxía, Facultade de Ciencias, Universidade da Coruña, Campus de Elviña, A Coruña 15071, Spain

**Keywords:** perfluoroalkyl acids, supramolecular cages, surfactant sequestration, host−guest chemistry, lipophilicity, environmental remediation

## Abstract

Perfluoroalkyl acids (PFAAs) are surfactants that rank among the most persistent and hazardous anthropogenic pollutants. Molecular cages have emerged as promising remediation agents, enabling the straightforward isolation and purification of PFAAs through selective binding and precipitation. However, critical gaps remain regarding (i) the degree of genuine selectivity for PFAAs over structurally analogous aliphatic surfactants, (ii) the structural features that govern cage–surfactant precipitation, and (iii) cage performance in mixed-surfactant systems. The absence of systematic investigations has impeded the rational design of cages with targeted selectivity and a limited understanding of their behavior in chemically complex environments. Herein, using one of the most efficient cages reported for PFAA removal, we address these questions and demonstrate that molecular cages do not intrinsically discriminate between fluorinated and aliphatic surfactants; instead, lipophilicity is the primary parameter controlling precipitation. Moreover, we show that surfactants in mixtures can be selectively isolated in an ordered fashion according to their lipophilicity.

## Graphical abstract

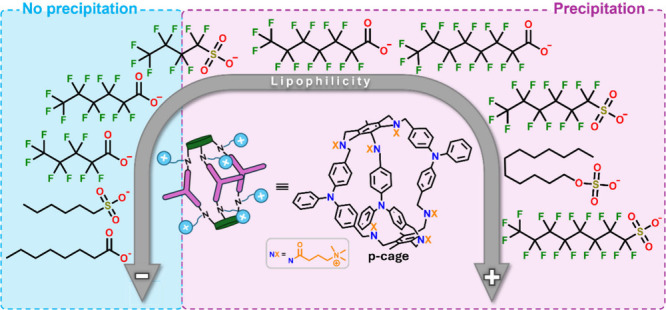

## Introduction

Surfactants, particularly anionic surfactants, are among the most widely utilized supramolecular units due to their exceptional surface-active properties.^1^ They are prevalent in diverse applications such as detergents, personal care products,^2^ pharmaceuticals,^3^ agrochemicals,^4^ and industrial cleaning formulations.^5^ However, despite their widespread utility, surfactants pose significant environmental risks by adversely affecting aquatic ecosystems, terrestrial organisms, and plant life.^6^ A notably hazardous class of anionic surfactants are perfluoroalkyl acids (PFAAs), distinguished by the exceptional strength and stability of their carbon–fluorine bonds.^7,8^ These compounds exhibit remarkable chemical stability, surface activity, and thermal resistance, which have made them indispensable in numerous industrial applications.^9,10^ Nevertheless, their environmental persistence^11,12^ and associated health risks^13^ have led to their classification as “forever chemicals”.^14^ Among PFAAs, perfluorooctanoic acid (PFOA) is the most representative, extensively used as a key emulsifier in the polymerization of polytetrafluoroethylene (PTFE),^15^ facilitating the production of high-performance materials for coatings, electronics, and aerospace industries.^16^ Despite these concerns, the global market for PFAAs remains strong, with PFOA alone currently valued at $10.17 billion and projected to exceed $11.5 billion by 2028.^17^

Recent studies have demonstrated that molecular cages provide a straightforward and effective strategy for the removal of perfluoroalkyl acids anions.^18–24^ These well-defined, discrete architectures feature enclosed internal cavities capable of interacting with guests,^25–29^ such as anionic surfactants,^30–32^ often inducing precipitation of the corresponding cage–guest complex.^18,21,22^ Despite these advances, significant knowledge gaps remain regarding the mechanistic factors that govern cage–PFAA interactions and the precipitation process. Key questions include the following. Q1: To what extent do the currently reported cages exhibit true selectivity for PFAAs over structurally analogous aliphatic surfactants? Q2: Which surfactant structural features primarily control cage–surfactant precipitation? And Q3: How do the cages behave in the presence of mixtures of surfactants? The absence of such analyses hinders the rational design of cages with targeted selectivity and limits our understanding of their performance in chemically complex environments.

The aim of this work is to address this critical knowledge gap and, in doing so, establish generalizable structure–selectivity principles to guide the design of next-generation cages capable of functioning effectively in complex, multicomponent aqueous environments. To this end, we employ our previously reported cage architecture (**p-cage**, Figure 1a), which is notable for its straightforward synthesis, high stability, fully organic composition, and exceptional performance, removing up to 19 PFAA anions per equivalent of cage. Importantly, we found that surfactant lipophilicity, i.e., the affinity of a molecule for a lipid environment relative to an aqueous one, is the key parameter controlling precipitation of the cage–surfactant complex and a lipophilicity difference of approximately one unit is sufficient to achieve selective separation between surfactants.

**Figure 1:**
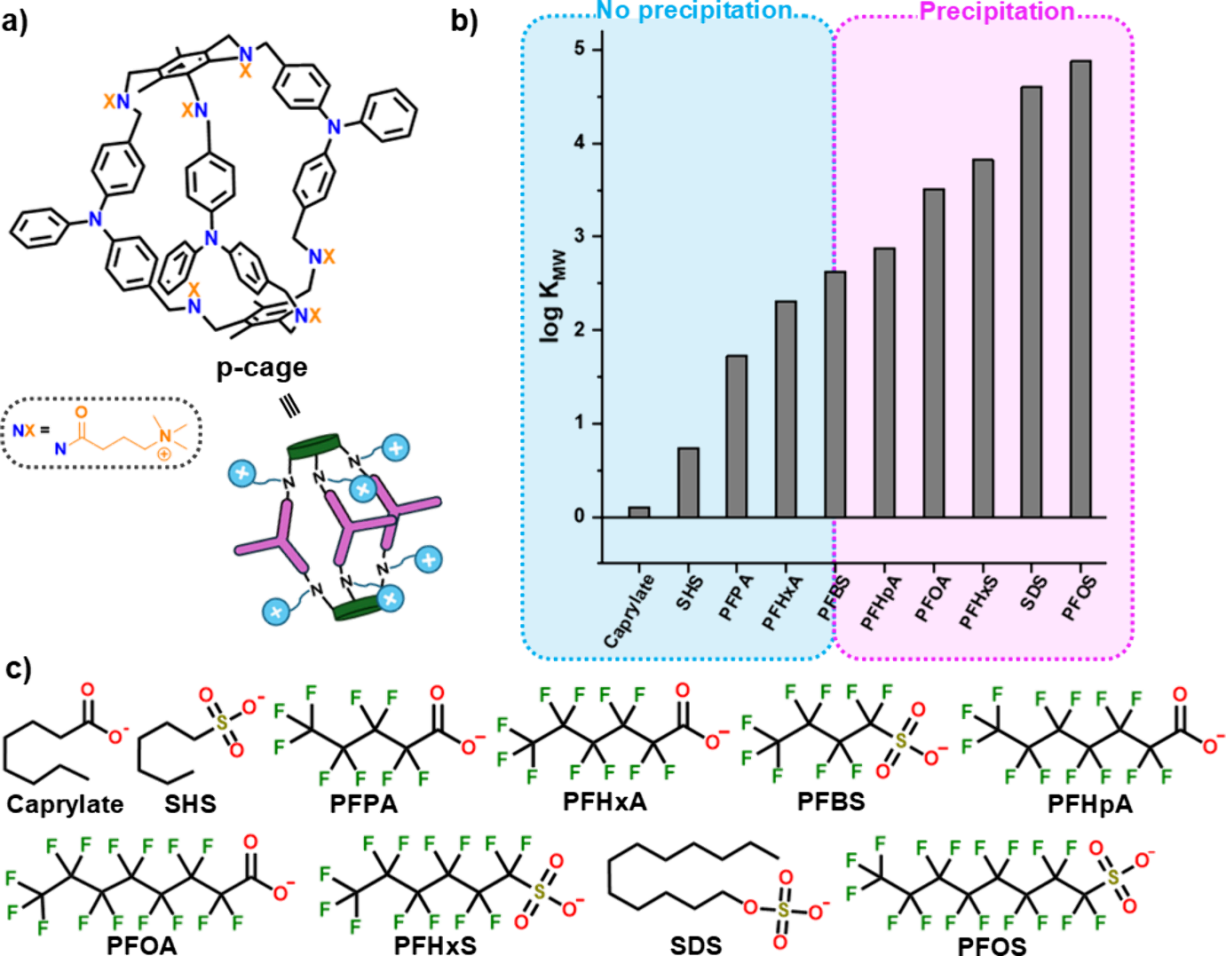
(a) Structure of the cationic molecular cage studied in this work, together with the cartoon favoring its representation. (b) Bar graph correlating the experimentally observed precipitation behavior (indicated by the shaded areas: no precipitation in blue; precipitation in pink) across a series of surfactants ordered by increasing log *K*_MW_ (partition coefficient between lipid membranes and water) values. (c) The specific molecular structures of the tested anionic surfactants are provided together with the names used throughout the document. Counterions are not shown for clarity.

## Experimental Methods

All reagents and solvents employed were commercially available and used as supplied without further purification, unless stated otherwise. We employed sodium salts of carboxylic surfactants and potassium salts for the sulfonates in all experiments. Proton nuclear magnetic resonance (^1^H NMR) and fluorine nuclear magnetic resonance (^19^F-NMR) spectra were measured on Bruker AVANCE III HD 300 Nuclear Magnetic Resonance spectrometer and were referenced relating to residual proton resonances in D_2_O (at 4.79 ppm) in ^1^H NMR. All chemical shifts (δ) values are given in parts per million. All ^19^F spectra are proton-decoupled, unless otherwise stated. The synthesis of **p-cage** was performed according to the procedures previously reported by our group. For the titrations, individual aqueous solutions of each surfactant were prepared at 1 mM and contained either NaBF_4_ (for perfluoroalkyl surfactants) or 1-ethyl-3-methylimidazolium chloride (IMIM-Cl, for aliphatic) as distinct internal standards. Then **p-cage** was added incrementally to each solution from a concentrated stock, and the resulting interaction was monitored by using NMR spectroscopy. Precipitation efficiency was assessed by monitoring the disappearance of the fluorine or proton signals from each surfactant. The ^1^H NMR experiments were carried out with a scan number of 48 and ^19^F NMR experiments with a scan number of 200.

## Results and Discussion

### Fluorinated vs Aliphatic Surfactants

To date, no studies have investigated the selectivity of PFAA removal using molecular cages in relation to anionic aliphatic surfactants.^19,22^ This is particularly important, because aliphatic surfactants are ubiquitous in industrial and environmental processes, often coexisting with PFAs in contaminated systems. Understanding the interaction between molecular cages and these surfactants is crucial for optimizing selective PFAA removal in real-world applications.

We previously reported that each **p-cage** molecule can efficiently interact with and precipitate 19 PFOA anions with both components fully regenerable through a low-energy, solvent-free acid treatment.^33^ Additionally, using perfluoropentanoic acid (PFPA) anions, we observed that PFAAs with shorter aliphatic tails interact with the **p-cage** but are not precipitated, assuming that the tail length was the key parameter controlling the precipitation process.

Building on the former observations, we now address Q1: whether the precipitation behavior is unique to PFAAs or also applies to aliphatic surfactants. Thus, an aqueous solution of sodium caprylate (1 mM), the aliphatic analogue of PFOA with an identical aliphatic chain length, was titrated with **p-cage** and monitored by ^1^H NMR (Figure S3). Interestingly, while the NMR spectrum showed evidence of interaction through peak shifts, no precipitation occurred, even at a 3-fold excess of cage (Figure 2). This suggests that **p-cage** may be a highly specific tool for PFAA removal.

**Figure 2:**
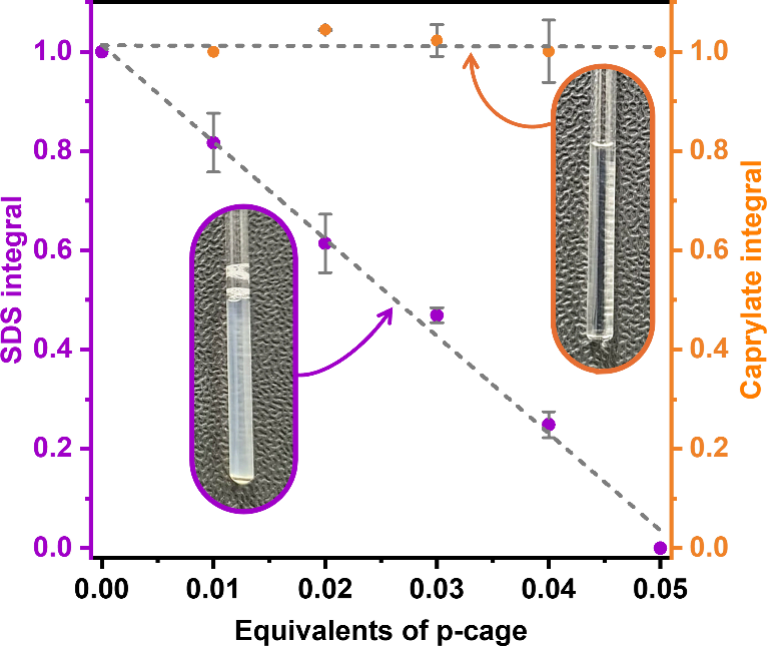
Comparison of the titration of SDS and caprylate with **p-cage**. Normalized surfactant integrals (SDS in purple, caprylate in orange), averaged over three independent replicates, are plotted as a function of **p-cage** equivalents added to individual surfactant solutions. Photographs of the corresponding NMR tubes are shown to illustrate the macroscopic precipitation of SDS upon cage addition, while caprylate remains fully soluble throughout the titration.

However, to further test this hypothesis, we conducted an additional experiment using another aliphatic surfactant, sodium dodecyl sulfate (SDS). Remarkably, SDS exhibited the same behavior as PFOA during the titration, fully precipitating upon the addition of a minimal amount, i.e., 0.05 equiv of **p-cage** (Figure S4). These results challenge our earlier hypotheses: (i) that hydrophobic tail length is the primary parameter governing precipitation, since PFOA and caprylate exhibit different behaviors despite having the same tail length, and (ii) that the **p-cage** acts as a selective precipitator for PFAAs. Consequently, our answer to Q1 is that for this cage (and likely for other reported cages as well) there is no clear specificity between perfluorinated and aliphatic anionic surfactants.

### Critical Micelle Concentration vs Lipophilicity

Having rejected the tail length as the parameter governing precipitation of the cage–surfactant complex, we now turn to Q2. Building on the results of the previous experiments and computational studies indicating that the cage may act as a cross-linking agent between surfactant micelles, we investigate whether the surfactant’s critical micelle concentration (CMC) is the primary factor driving precipitation. To test this, we compared the cage’s behavior with three surfactants: (i) PFOA (CMC = 35 mM), (ii) SDS (CMC = 8 mM), and (iii) potassium nonafluoro-1-butanesulfonate (PFBS, CMC = 22 mM). As previously mentioned, the **p-cage** efficiently precipitated SDS and PFOA, with complete removal observed in NMR experiments upon adding only 0.05 equiv of cage (Figures S4 and S9). If the CMC were the sole determinant of precipitation, we would expect PFBS to behave similarly to PFOA, since its CMC lies between those of the former surfactants. However, the results from the PFBS titration were unexpected, as it only partially precipitated (Figure S5). For instance, with 0.05 equiv of **p-cage** (i.e., the amount required for full precipitation of the other surfactants), only about 45% of PFBS precipitated (as determined from the surfactant integrals). Moreover, even after adding 0.5 equiv of **p-cage**, PFBS was not fully removed (Figure S6).

Under realistic conditions, surfactant concentrations can vary substantially, potentially affecting precipitation behavior, as this process is governed by equilibrium thermodynamics. Accordingly, we investigated the effect of the initial surfactant concentration on the precipitation efficiency of **p-cage**. A 2 mM solution of PFBS, twice the concentration previously examined, was prepared and treated with up to 0.05 equiv of **p-cage**. Partial precipitation was observed, analogous to the behavior at lower equivalents in the 1 mM experiment. Notably, the residual surfactant concentration converged to the same final value measured in the 1 mM system (Figure S7). These findings indicate that although a higher initial concentration enables a greater absolute amount of surfactant to precipitate, the system ultimately converges to an identical equilibrium concentration, consistent with thermodynamically controlled precipitation

To confirm that CMC is not a key factor in surfactant precipitation, we examined the effect of **p-cage** addition on two PFAA surfactants, perfluorohexanoic acid (PFHxA) and perfluoroheptanoic acid (PFHpA), which have similar CMCs (110 mM and 103 mM, respectively).^34,35^ In this case, PFHpA exhibited the same behavior as SDS and PFOA, fully precipitating upon titration with 0.05 equiv of **p-cage**, as previously observed by our group. In contrast, PFHxA does not precipitate upon addition of 0.05 equiv of **p-cage**, with its peak integrals remaining constant throughout the titration (Figure S8). Together, these results indicate there is no clear correlation between CMC values and precipitation behavior.

Our final hypothesis, before considering the complex interplay of factors governing precipitation, was that surfactant lipophilicity could be the key factor driving this phenomenon. Lipophilicity refers to the affinity of a molecule for a lipid environment relative to an aqueous one. For charged molecules, lipophilicity can be quantified using the partition coefficient between lipid membranes and water, denoted as K_MW_ and typically expressed as log *K*_MW_. Higher log *K*_MW_ values indicate greater lipophilicity, reflecting a stronger affinity of the compound for nonpolar solvents.

Thus, we compiled the results from the previous experiments and incorporated new data to generate the graph shown in Figure 1b, where surfactants are ordered based on their lipophilicity.^36^ Interestingly, the graph reveals a clear distinction between two regions: surfactants with a log *K*_MW_ higher than 2.63 precipitate, regardless of their chemical nature, while those with a lower log *K*_MW_ interact with the cage, as evidenced by chemical shift perturbations upon titration with **p-cage**, but do not precipitate. This indicates the boundary between precipitation and a mere interaction. These findings directly address the Q2 question raised in the introduction, establishing lipophilicity as the dominant factor controlling the surfactant precipitation. However, once a surfactant’s lipophilicity surpasses the threshold required for precipitation, each equivalent of cage consistently precipitates the same amount of surfactant, regardless of lipophilicity, as approximately 0.05 equiv of **p-cage** were sufficient to fully remove the surfactant in all cases.

### Competitive Experiments in Binary Surfactant Mixtures

We then move to answer Q3 and hypothesized that competition experiments, where **p-cage** faces two different surfactants simultaneously, would be ideal for: (i) determining if one surfactant interferes with the other due to the formation of mixed micelles, (ii) confirming that lipophilicity is the primary factor driving precipitation, and (iii) evaluating the potential of using the cage to isolate individual surfactants from mixtures.

#### (i) Influence of Mixed Aggregation

When two or more surfactants are present, they naturally tend to coaggregate, forming mixed micelles. The formation of mixed micelles is governed by regular solution theory, where the interaction parameter (β) quantifies the nature of the surfactants’ interactions. A negative β value indicates synergistic interactions, suggesting that the surfactants interact favorably, enhancing micelle formation. In contrast, a positive β value points to antagonistic interactions, where the surfactants repel each other, potentially reducing the micelle formation efficiency. Thus, negative or near-zero β values in a surfactant mixture suggest a tendency to form mixed micelles, while positive values indicate a preference for the surfactants to form independent aggregates.^37^ In general, anionic surfactants with a single aliphatic chain exhibit β values that are either negative or close to zero, suggesting a tendency to form mixed micelles. The same holds true for mixtures of fluorinated surfactants.^38^ However, when aliphatic and fluorinated surfactants are mixed, the β values often shift to positive values. This shift is due to the differences in hydrophobicity and structural incompatibilities between the aliphatic and fluorinated tails, which can hinder the formation of stable mixed micelles and lead to more independent aggregation of the surfactants.^39–41^

To investigate whether coaggregation influences surfactant removal through precipitation, we began by studying a simple system: a mixture of two surfactants: one that the cage does not precipitate (caprylate) and one that does (PFOA). In this case, the two surfactants have a low tendency to form mixed micelles due to the differing nature of their tails, as explained earlier. Thus, an equimolar solution of both surfactants (1 mM each) in D_2_O was then titrated with **p-cage**, with ^1^H NMR used to monitor caprylate and ^19^F-NMR to track PFOA. The results show that after the addition of 0.05 equiv of **p-cage**, only PFOA precipitates, while caprylate remains in solution and does not precipitate, even after an additional 0.05 equiv of cage are added (Figure S12). This demonstrates excellent orthogonality in the precipitation behavior of **p-cage**.

Building on the success of the previous experiment, we advanced to the next step and evaluated two surfactants with a higher tendency to form mixed micelles due to fluorine–fluorine interactions. One of these surfactants, PFOA, showed precipitation in earlier experiments, while the other, PFHxA, did not. Remarkably, when an equimolar mixture of the two was titrated with **p-cage**, only PFOA precipitated after the addition of 0.05 equiv (Figure S13). These results show that the cage’s precipitation behavior, initially observed for individual surfactants, is retained even when surfactants are present as mixtures capable of forming mixed micelles, thus directly answering the question of how such systems behave in complex anionic surfactant mixtures.

#### (ii) Role of Lipophilicity Differences

Building on the previous results, which rule out limitations related to mixed micelle formation, we now shift our focus from micelle formation to examining the differences in lipophilicity between the surfactants. We therefore propose to study three mixed surfactant systems with progressively diminishing differences in lipophilicity. In all cases, the individual surfactants exhibit precipitation when tested separately, allowing a direct comparison of how lipophilicity governs selective versus simultaneous precipitation.

First, we examined the PFOA/SDS system (Figure 3a), which displays a large lipophilicity difference (Δ log *K*_MW_ = 1.1). Upon incremental addition of **p-cage**, SDS precipitated first, achieving complete removal with only 0.04 equiv of cage. At this stage, only a minor fraction of PFOA (≈10%) was co-precipitated (Figure 3a). This high selectivity is particularly noteworthy, as PFOA is one of the most common PFAs, and SDS is a widely used aliphatic surfactant. The ability to perform hierarchical precipitation of both surfactants, PFOA and SDS, enabling their orthogonal separation, showcases the cage’s remarkable precision in targeting specific contaminants and underscores its potential for highly selective environmental and industrial applications.

**Figure 3:**
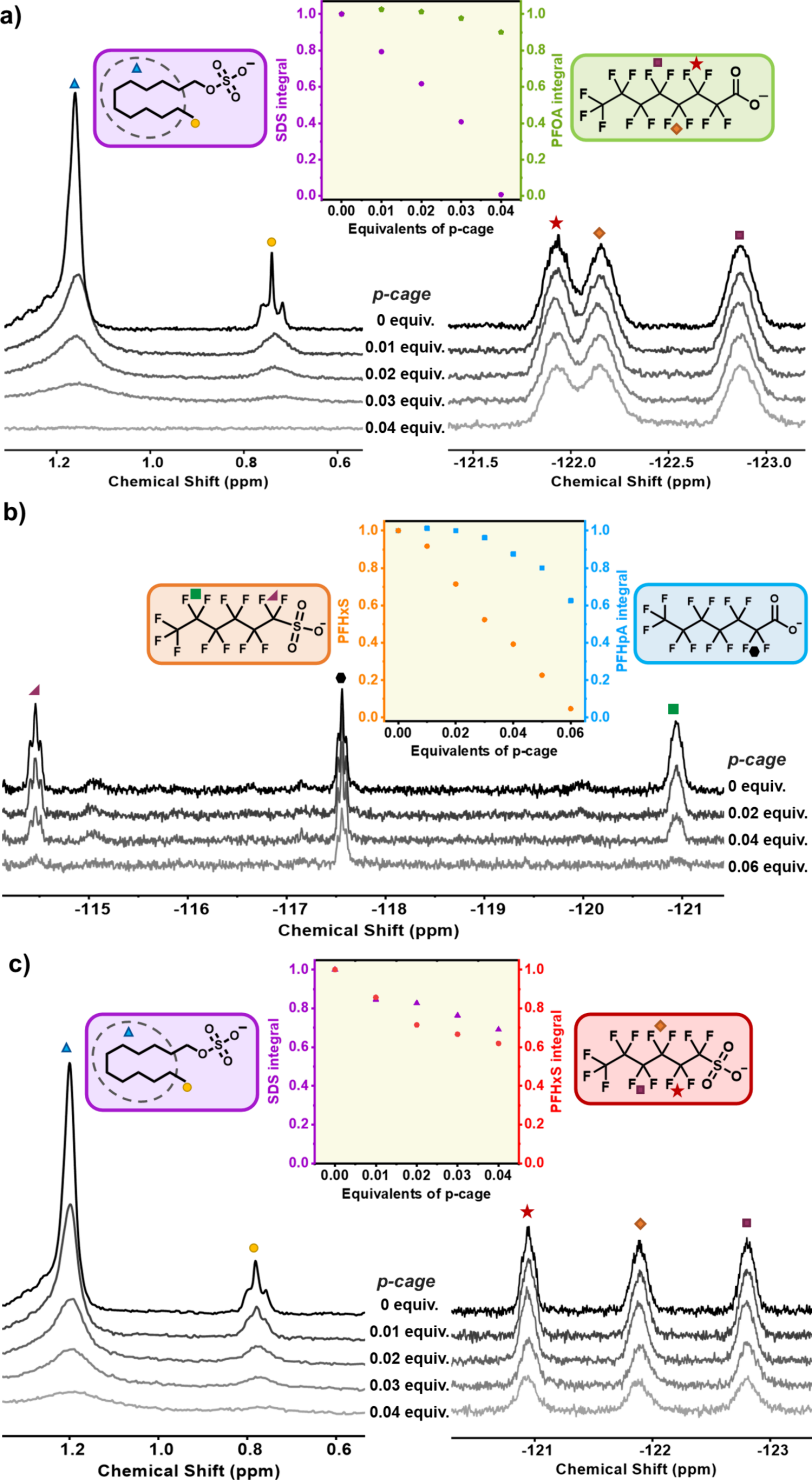
Competitive experiments in binary surfactant mixtures probing the role of lipophilicity in hierarchical precipitation. (a) Equimolar PFOA/SDS mixture: Upon **p-cage** addition, ^1^H NMR spectra show rapid SDS decay, with complete removal at 0.04 equiv, while PFOA remains largely in solution, as shown by ^19^F NMR. Normalized integrals (SDS, purple; PFOA, green) are plotted versus **p-cage** equivalents. (b) Equimolar PFHpA/PFHxS mixture: Both surfactants decrease upon **p-cage** addition, with PFHxS preferentially precipitating and fully removed at 0.06 equiv. At this point, approximately 65% of PFHpA remains in solution. Normalized integrals (PFHpA, blue; PFHxS, orange) are shown versus **p-cage** equivalents. (c) Equimolar SDS/PFHxS mixture: NMR spectra show the simultaneous precipitation of both surfactants upon **p-cage** addition. Normalized integrals (SDS, purple; PFHxS, orange) decrease similarly; when 39% of PFHxS is removed, 31% of SDS also precipitates.

Next, we combined two precipitating fluorinated surfactants, PFHpA and PFHxS, which exhibit a smaller lipophilicity difference (Δ log *K*_MW_ = 0.95). Upon titration with **p-cage**, both surfactants eventually precipitated, with PFHxS, bearing a sulfonate group, precipitating first (Figure 3b). However, the selectivity was markedly reduced compared with the SDS/PFOA system. Approximately 35% of PFHpA co-precipitated alongside PFHxS, and an additional 0.05 equiv of cage were required to achieve complete removal of the remaining carboxylate surfactant. This attenuation in selectivity correlates directly with the reduced lipophilicity difference, indicating that a sufficiently large Δlog K_MW_ is required to establish a clear hierarchy of precipitation.

Finally, based on these observations, we hypothesized that an even smaller lipophilicity difference would lead to essentially identical behavior for both surfactants. Accordingly, we selected SDS and PFHxS, for which Δ log *K*_MW_ = 0.8, and an equimolar mixture of the two surfactants was titrated with **p-cage** (Figure 3c). As expected, precipitation occurred nearly simultaneously: when 39% of PFHxS had precipitated, 31% of SDS had also been removed from solution.

Collectively, these systematically paired comparisons demonstrate that the precipitation behavior is not dictated by a single structural feature or by preorganized aggregation phenomena. Instead, the molecular cage operates as a quantitative lipophilicity sensor, with the precipitation response being governed by the relative lipophilicity of the resulting host–guest complexes.

### Final Experiment: Separation of a Three-Component Mixture

Following the establishment of both the general precipitation threshold (log *K*_MW_ > 2.87) and the required lipophilicity differential (Δ log *K*_MW_ > 0.95) for sequential separation, we sought to demonstrate the functional utility of the **p-cage** in resolving a truly complex, multicomponent surfactant mixture. This final experiment definitively validates that the precipitation mechanism is a hierarchical process strictly governed by the quantitative lipophilicity of the surfactants.

The mixture was composed of three surfactants, each at 1.0 mM: SDS, PFOA, and SHS whose lipophilicity is below the critical precipitation threshold. These guests represent a range of lipophilicities that are, in principle, amenable to sequential separation by the cage. The expected order of precipitation, based on our mechanistic understanding, was SDS, followed by PFOA, with SHS remaining in solution.

The results of the competitive titration were highly resolved and validated the lipophilicity-driven mechanism. SDS, possessing the highest lipophilicity in the mixture (approximately 4.61), was the first component to be completely removed from the solution. The complete precipitation of SDS was achieved upon the addition of 0.04 equiv of the **p-cage** (40 μM). At this stage, the concentration of the second most lipophilic guest, PFOA, remained largely in solution, as indicated by the normalized integral of the PFOA ^19^F NMR signal, corresponding to 92 ± 4% of the initial value (Figure 4c). This indicates that the cage exhibits an unequivocal preference for the most lipophilic guest under competitive conditions, successfully overcoming the challenge of simultaneous precipitation. Complete PFOA removal was subsequently achieved with further cage addition, requiring 0.10 equiv of **p-cage** (Figure 4b,c) reinforcing the notion of a robust, sequential, and selective precipitation process dictated by the subtle differences in log K_MW_.

**Figure 4:**
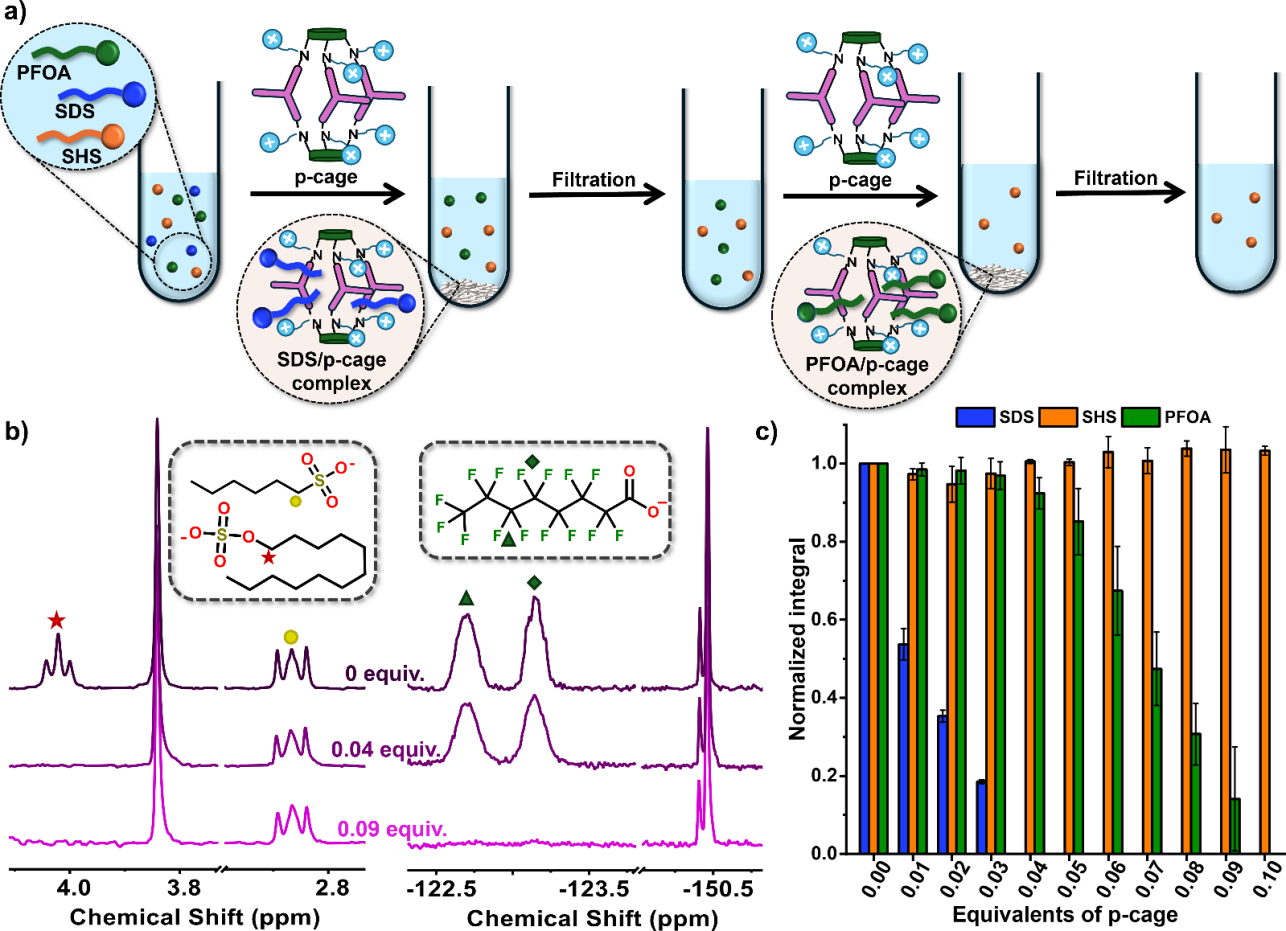
(a) Scheme of the sequential separation process where **p-cage** is added to a mixture of the three surfactants: PFOA (green), SDS (blue), and SHS (orange). Upon addition, SDS precipitates first and is removed by filtration. A second addition with **p-cage** induces precipitation of PFOA, which is also isolated by filtration. SHS remains in solution throughout, as it does not precipitate under these conditions. (b) Expanded views of the diagnostic regions in the ^1^H NMR (left) and ^19^F NMR (right) spectra for a mixture of SDS, 6C, and PFOA (1 mM each), recorded with 1-ethyl-3-methylimidazolium chloride and NaBF_4_ as internal standards. After the addition of 0.04 equiv of **p-cage** (40 μM), SDS signals disappear while SHS and PFOA remain unaffected. Following the addition of 0.1 equiv, only SHS signals are observed, confirming complete removal of PFOA from solution. (c) Bar chart showing the normalized integrals of representative NMR peaks for each surfactant as a function of **p-cage** equivalents. SDS is fully removed at 0.04 equiv (40 μM), PFOA at 0.1 equiv (100 μM), while SHS remains soluble across all conditions. Data represent the mean of three replicates.

Remarkably, the short-chain surfactant, SHS, remained fully dissolved throughout the entire titration, even in the presence of a substantial excess of the cage. This final observation confirms the stringent adherence of the system to the previously established precipitation threshold. Ultimately, this demonstrates that the cage is a highly effective supramolecular tool capable of high-resolution separation of complex mixtures, with the recovery order strictly determined by the guest’s lipophilicity. Moreover, both the cage and the isolated surfactants can be readily recovered using previously established methods from our group, providing a pathway to a sustainable, closed-loop separation process.

## Conclusions

In summary, we have used a promising fully organic molecular cage, **p-cage**, to gain valuable insights into the phenomenon of molecular-cage-induced surfactant precipitation. The key conclusions are as follows: (i) Lipophilicity, quantified as log *K*_MW_, is the primary factor governing surfactant precipitation. Our results establish a critical precipitation threshold at log *K*_MW_ > 2.63, below which surfactants interact with the **p-cage** without precipitating. (ii) PFAS and aliphatic surfactants precipitate similarly with the **p-cage**, making them indistinguishable in terms of precipitation behavior. (iii) The formation of mixed micelles does not interfere with surfactant precipitation. (iv) A minimum lipophilicity differential (Δ log *K*_MW_ > 0.95) is necessary to achieve high-fidelity, sequential separation of surfactants. As a result, a three-component mixture comprising SDS, PFOA, and SHS has been successfully isolated, demonstrating that the cage functions as a hierarchical molecular selector, precipitating anionic surfactants strictly based on their lipophilicity.

We believe this knowledge will pave the way for refining molecular cage structures, enabling them to precipitate fewer lipophilic surfactants and lower the selectivity threshold. This could enhance the separation of complex surfactant mixtures, ultimately reducing production costs and minimizing the environmental impact.

## Supplementary Material



## Notes

Experimental methods, host–guest experimental results, and titrations and competition experimental results (PDF)
