# Supplementary material for: Decoding the Principles Governing Molecular Cage Precipitation of Aliphatic and Perfluoroalkyl Acids (PFAAs)
Source: ACS Appl Mater Interfaces. 2026 Mar 10;18(11):16609–16. doi: 10.1021/acsami.6c01610 (PMC13022805; doi:10.1021/acsami.6c01610)
Supplement: Supplementary file 1 [file am6c01610_si_001.pdf]

Supporting Information

Decoding the Principles Governing Molecular Cage Precipitation of Aliphatic and Perfluoroalkyl Acids (PFAAs)

María Pérez-Ferreiro[1], Alejandro Criado[1,], and Jesús Mosquera[1,]**

[1] CICA−Centro Interdisciplinar de Química e Bioloxía, Facultade de Ciencias, Universidade da Coruña, Campus de Elviña, 15071, A Coruña, Spain

*Corresponding Authors: Alejandro Criado a.criado@udc.es; Jesús Mosquera j.mosquera1@udc.es

TABLE OF CONTENTS

1. General methods 3

2. Synthesis of p-cage 3

3. Titration of individual surfactant solutions 4

3.1. Aliphatic surfactants 4

3.2. Perfluoroalkyl surfactants 7

4. Titration of paired mixtures of surfactants 12

4.1. PFOA – SDS mixture 12

4.2. Caprylate – PFOA mixture 13

4.3. PFHxA – PFOA mixture 13

5. Titration of three-component mixture 14

5.1. PFOA – SDS – SHS mixture 14

1. General methods

Proton nuclear magnetic resonance (^{1}H-NMR) and fluorine nuclear magnetic resonance (^{19}F-NMR) spectra were measured on Bruker AVANCE III HD 300 Nuclear Magnetic Resonance spectrometer and were referenced relating to residual proton resonances in D_2O (at 4.79 ppm). All chemical shifts (δ) values are given in parts per million. All coupling constants are quoted in Hz. All ^{13}C and ^{19}F spectra are proton decoupled unless otherwise stated. The ^{1}H-NMR experiments were carried out with a scan number of 48 and ^{19}F-NMR experiments with a scan number of 200. Every titration was done using a concentration of 1 mM of surfactant. For each addition of cage, the corresponding equivalents were added from a concentrated stock in D_2O.

2. Synthesis of p-cage

The synthesis of **p-cage** was performed according to the procedures previously reported by our group (Figure S1).[1]

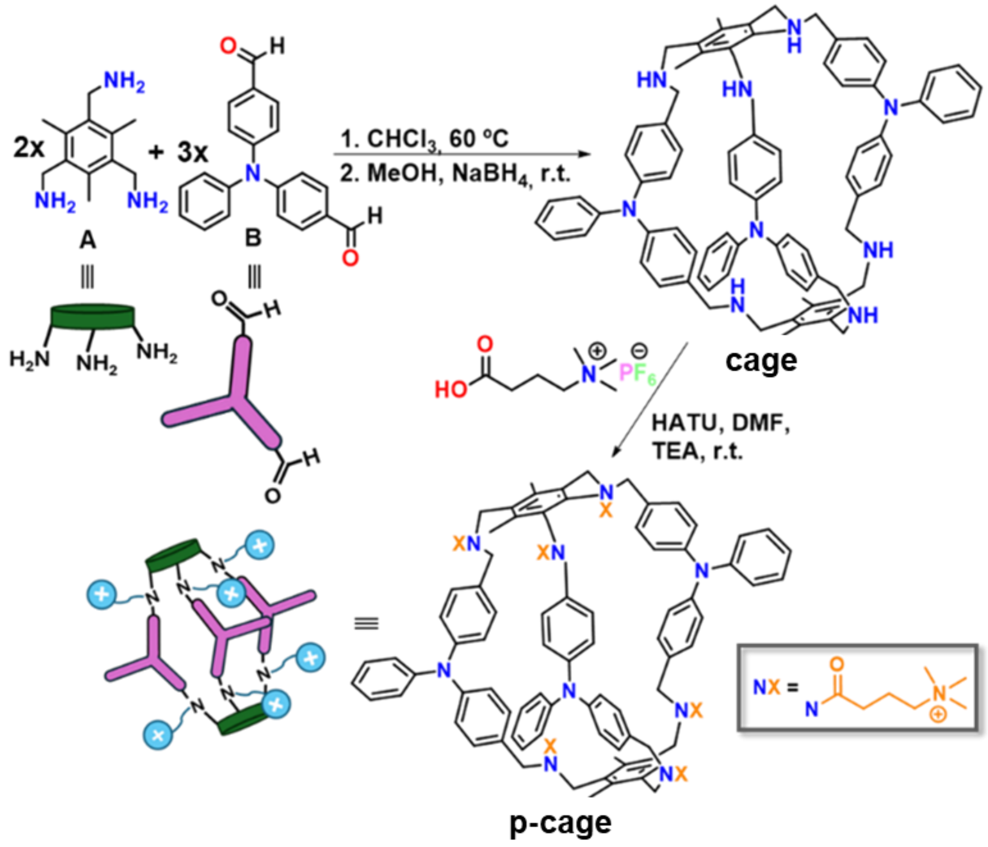

Figure S1. *Schematic representation of the synthesis of **p-cage**.*

3. Titration of individual surfactant solutions

3.1. Aliphatic surfactants

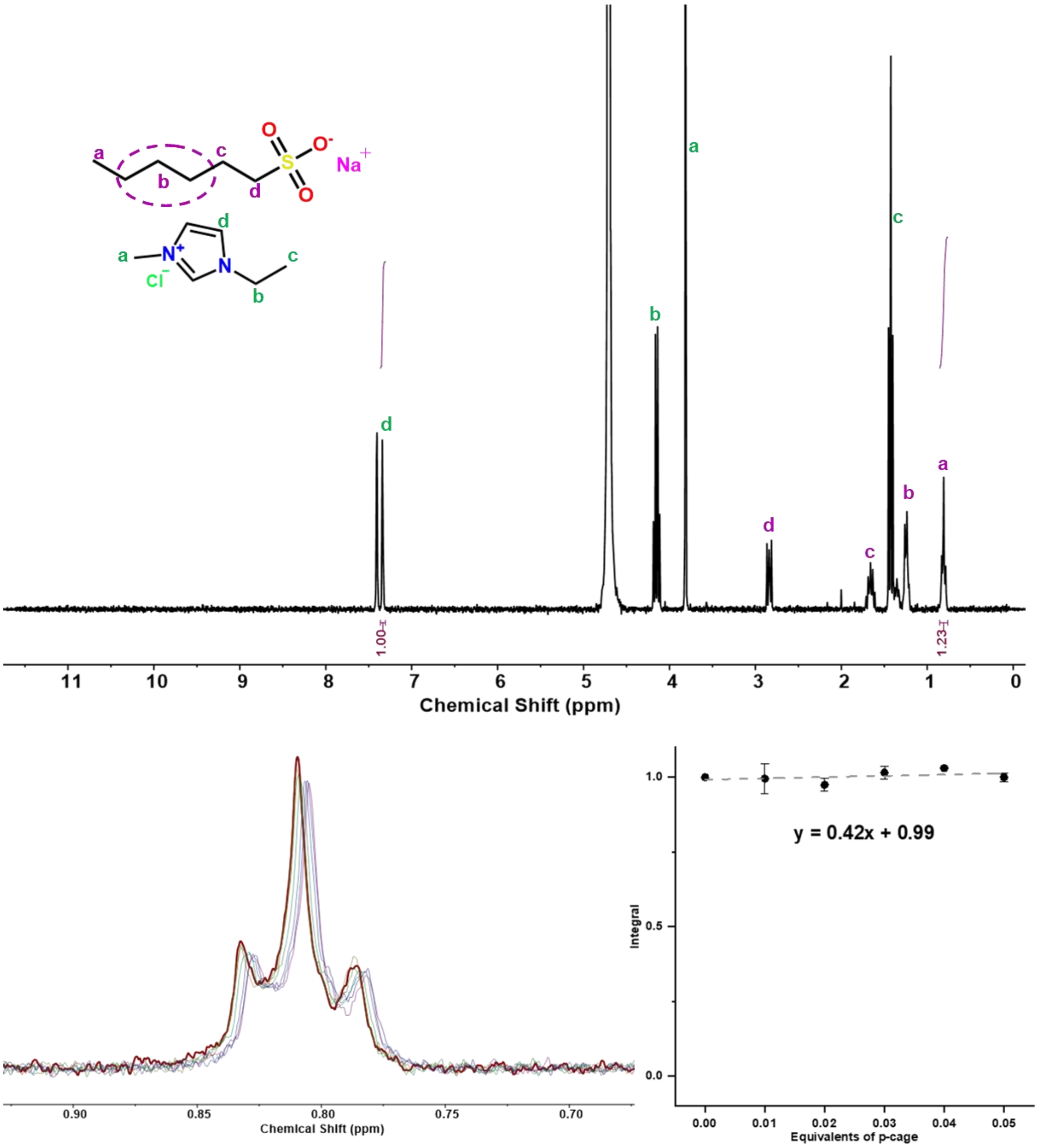

Figure S2. *^{1}H-NMR (300 MHz,298 K) of 1 mM **SHS** and 3 mM reference. Zoom in the methyl terminal group from the surfactant after the addition of up to 0.05 equivalents of **p-cage**. Zoom in the surfactant's terminal methyl group signal shows interaction but no signal decay. Graphical representation of the integral of surfactants signal vs the equivalents of **p-cage** added.*

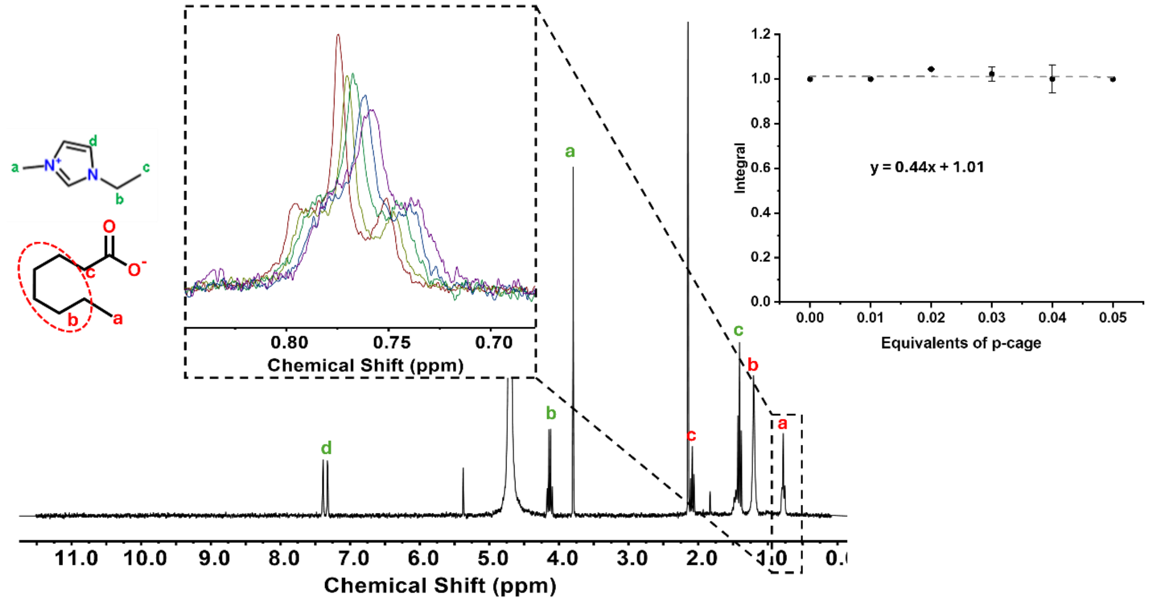

***Figure S3**. ^{1}H-NMR (300 MHz,298 K) of 1 mM caprylate and 1 mM reference. Zoom in the methyl terminal group from the surfactant after the addition of up to 0.05 equivalents of **p-cage**. Zoom in the surfactant's terminal methyl group signal shows interaction but no signal decay. Graphical representation of the integral of surfactants signal vs the equivalents of **p-cage** added.*

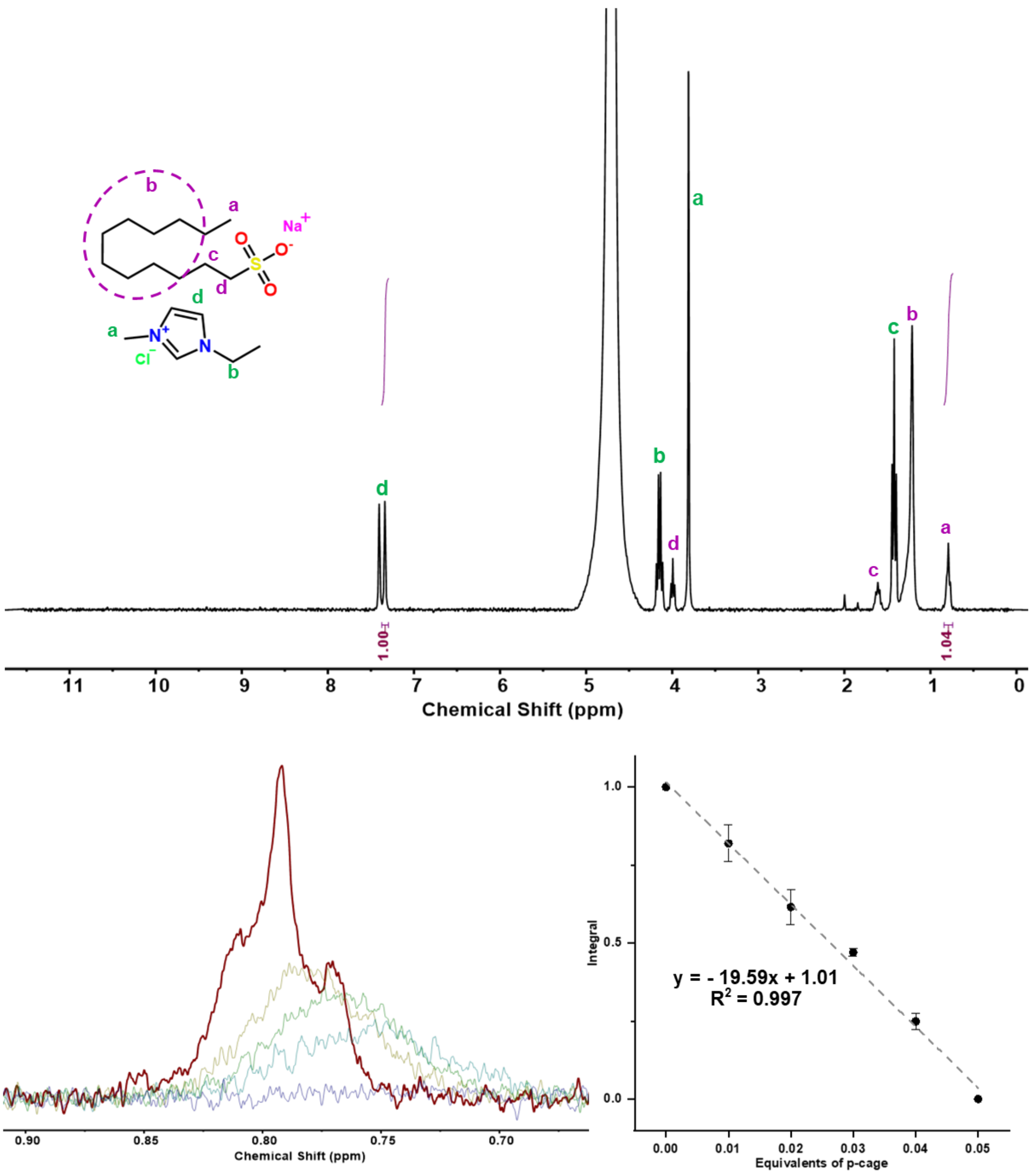

Figure S4. *^{1}H-NMR (300 MHz, 298 K) of SDS (1 mM) and reference (3 mM). Zoom in the methyl terminal group from the surfactant after the addition of up to 0.05 equivalents of* ***p-cage****. Zoom in the surfactant's terminal methyl group signal shows signal decay until complete elimination. Graphical representation of the integral of surfactants signal vs the equivalents of* ***p-cage*** *added, showing the elimination of around 20 surfactant molecules.*

3.2. Perfluoroalkyl surfactants

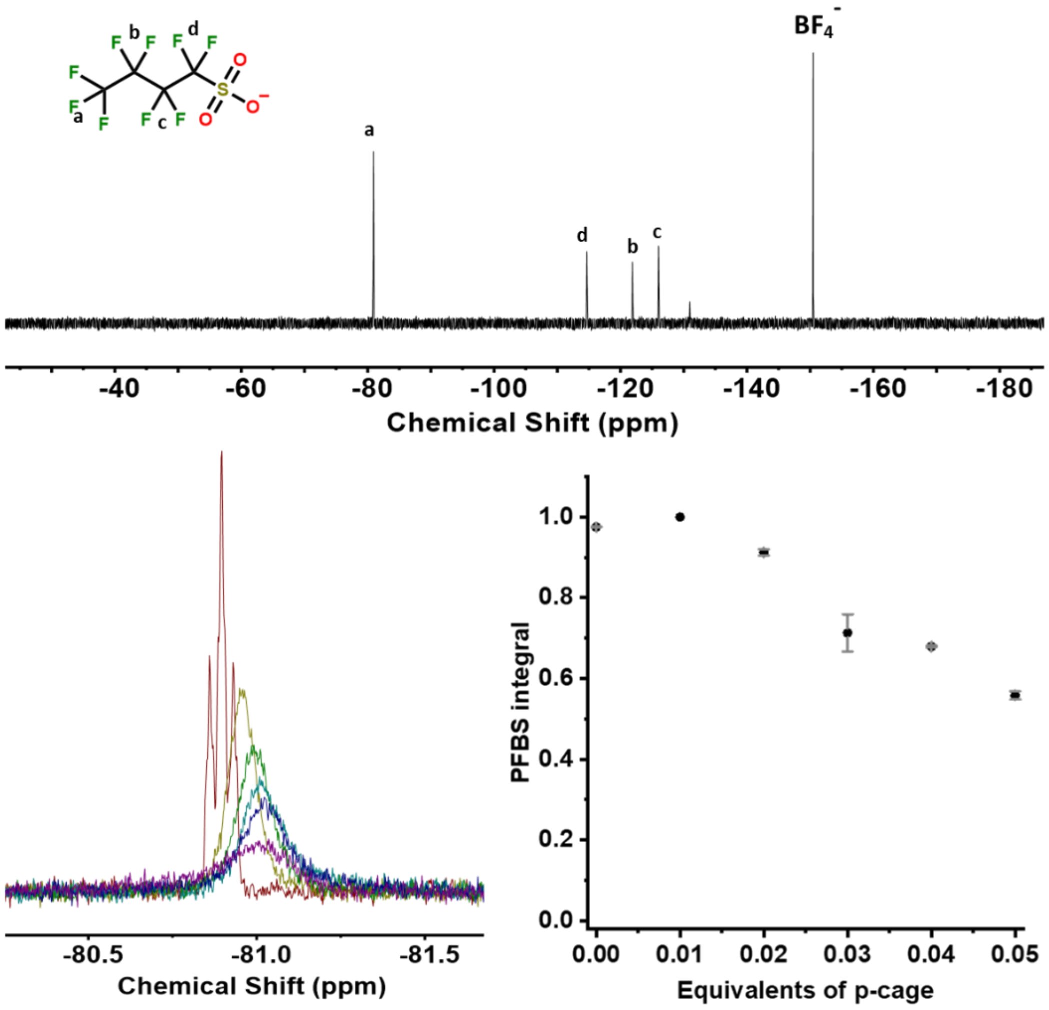

Figure S5. ^{19}F-NMR (282 MHz, D_2O) titration of PFBS (1 mM) and $NaBF_4$ (1 mM) with **p-cage.** Zoom in the surfactant's signals, after the addition of up to 0.05 equivalents of **p-cage**. Graphical representation of the integral of surfactants signal vs the equivalents of **p-cage** added, showing the elimination of around 45% of PFBS.

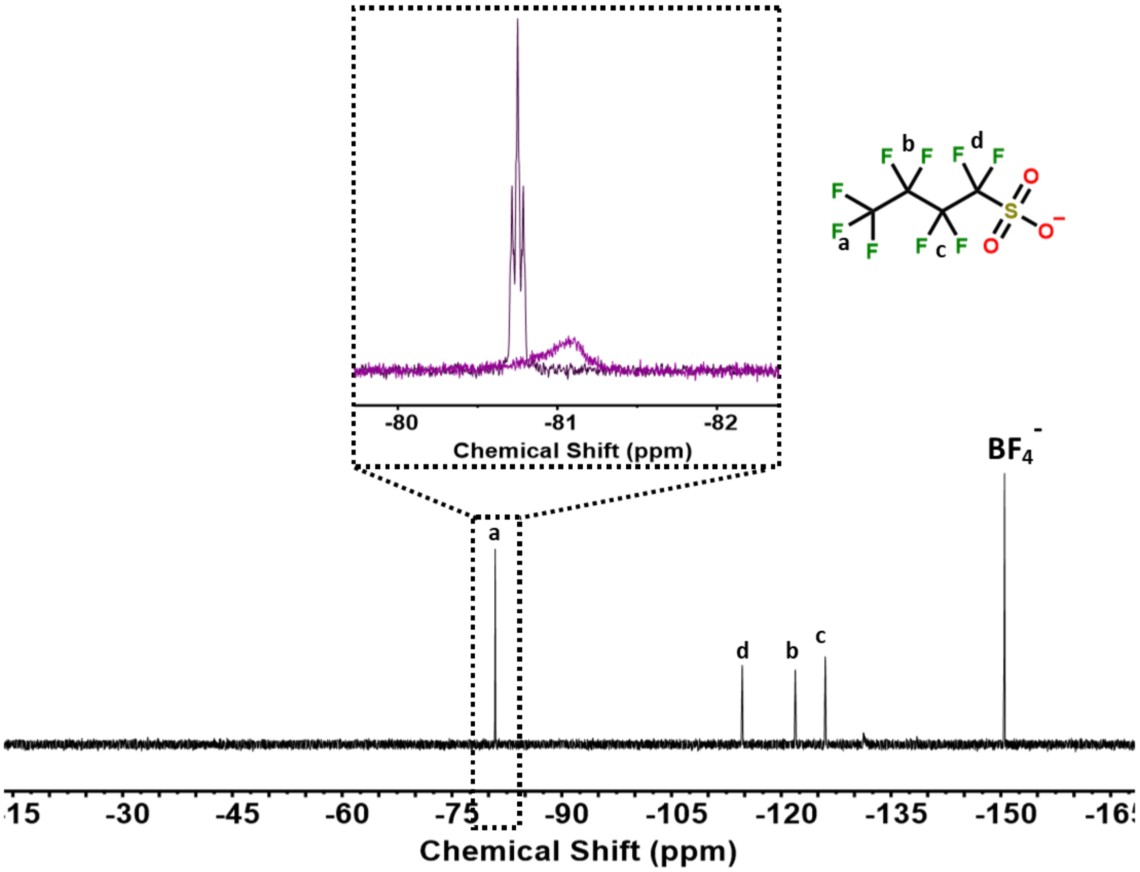

Figure S6. *^{19}F-NMR (282 MHz, D_2O) titration of potassium nonafluoro-1-butanesulfonate (1 mM) and $NaBF_4$ (1 mM) with **p-cage.** Zoom in the surfactant signal after the addition of 0.5 equivalents of **p-cage**.*

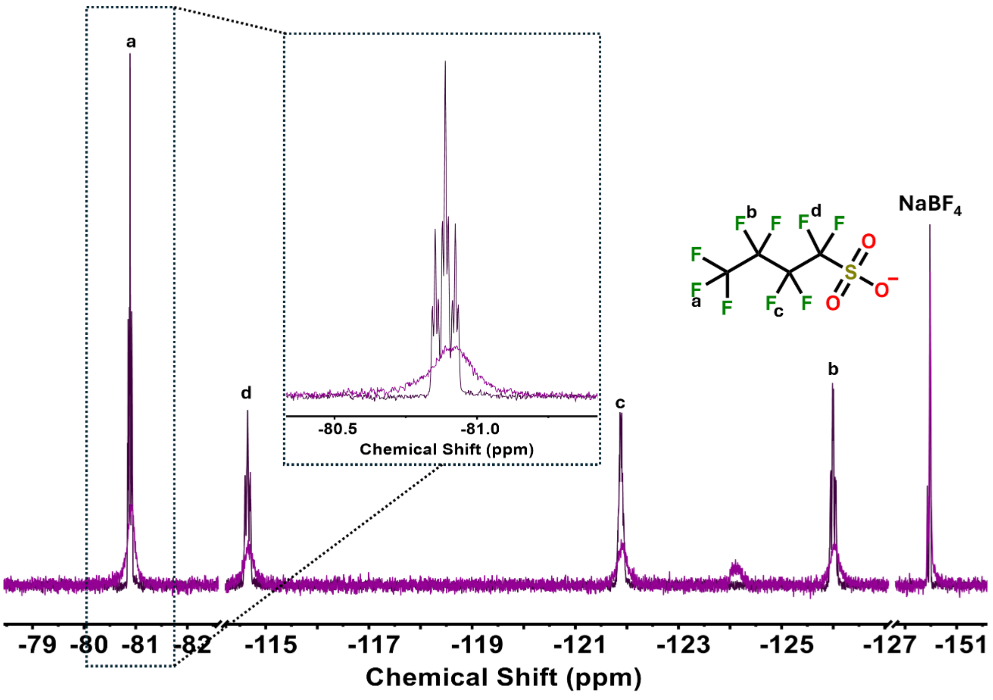

Figure S7. *^{19}F-NMR (282 MHz, D_2O) titration of potassium nonafluoro-1-butanesulfonate (2 mM) and $NaBF_4$ (1 mM) with **p-cage.** Zoom in the surfactant signal after the addition of 0.05 equivalents of **p-cage**.*

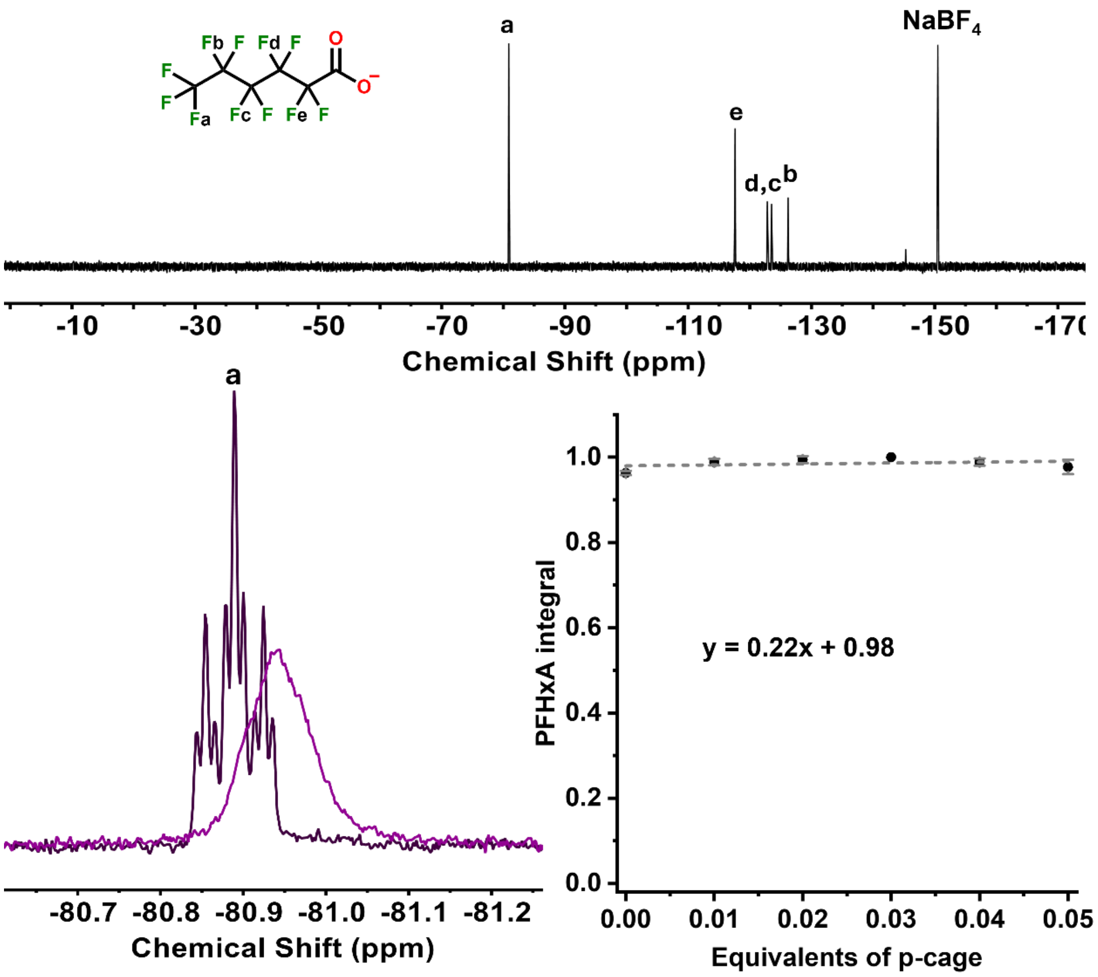

Figure S8. *^{19}F-NMR (282 MHz, D_2O) titration of PFHxA (1 mM) and $NaBF_4$ (1 mM) with* ***p-cage****. Zoom in the surfactant's signals, showing displacement and broadness of the surfactants signal due to interaction with the added cage (0.05 equivalents). The graph represents the constant integral of the terminal -CF_3 from PFHxA upon addition of* ***p-cage****.*

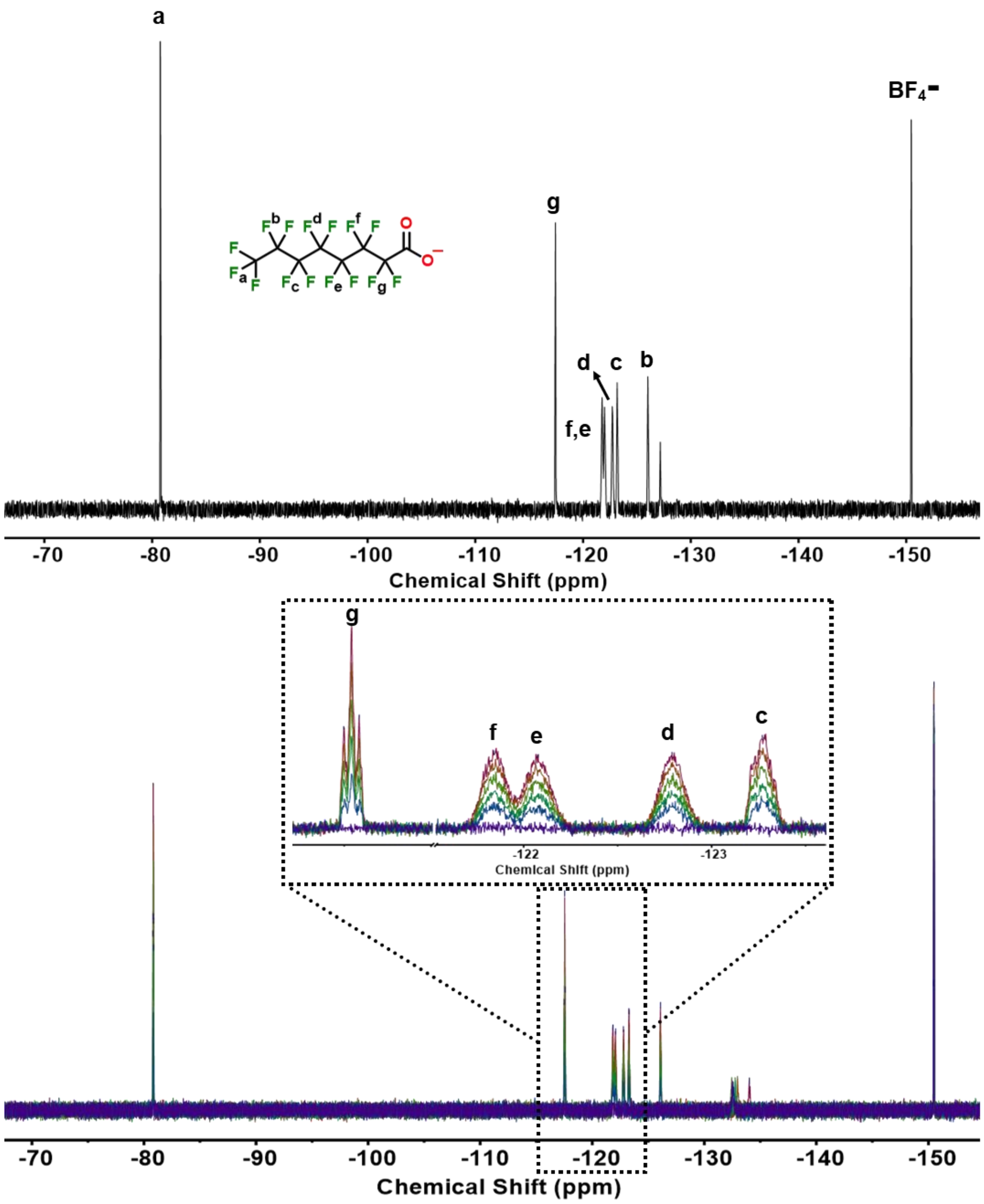

Figure S9. *^{19}F-NMR (282 MHz, D_2O) of PFOA (1 mM) and $NaBF_4$ (0.5 mM) in D_2O (above) and superimposed with the obtained spectrum after the addition of up to 0.05 equivalents of* ***p-cage****. Zoom in the surfactant's signals, showing signal decay until complete removal.*

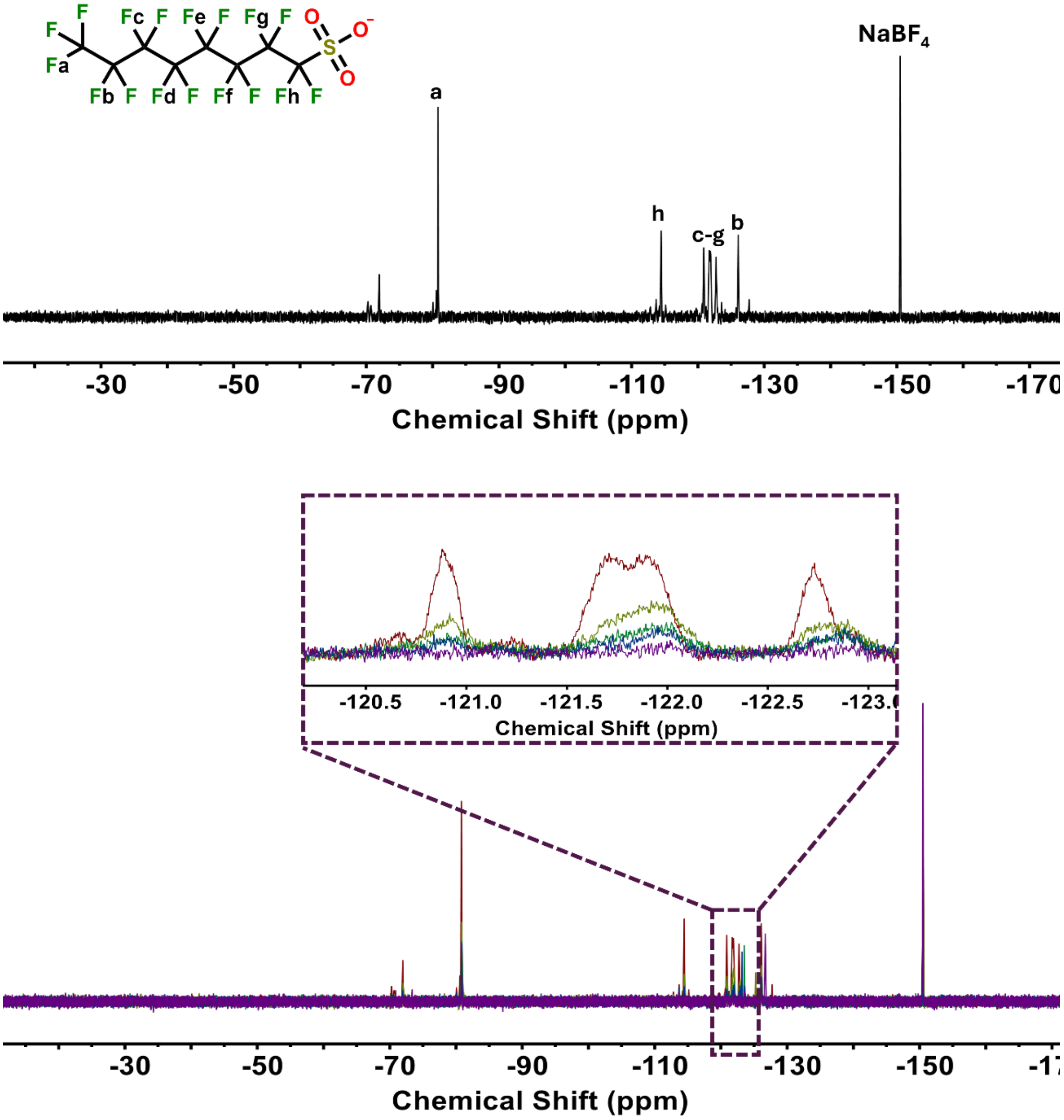

Figure S10. *^{19}F-NMR (282 MHz, D_2O) of PFOS (1 mM) and $NaBF_4$ (1 mM) in D_2O (above) and superimposed with the obtained spectrum after the addition of up to 0.08 equivalents of* ***p-cage****. Zoom in the surfactant's signals, showing signal decay until complete removal.*

4. Titration of paired mixtures of surfactants

4.1. PFOA – SDS mixture

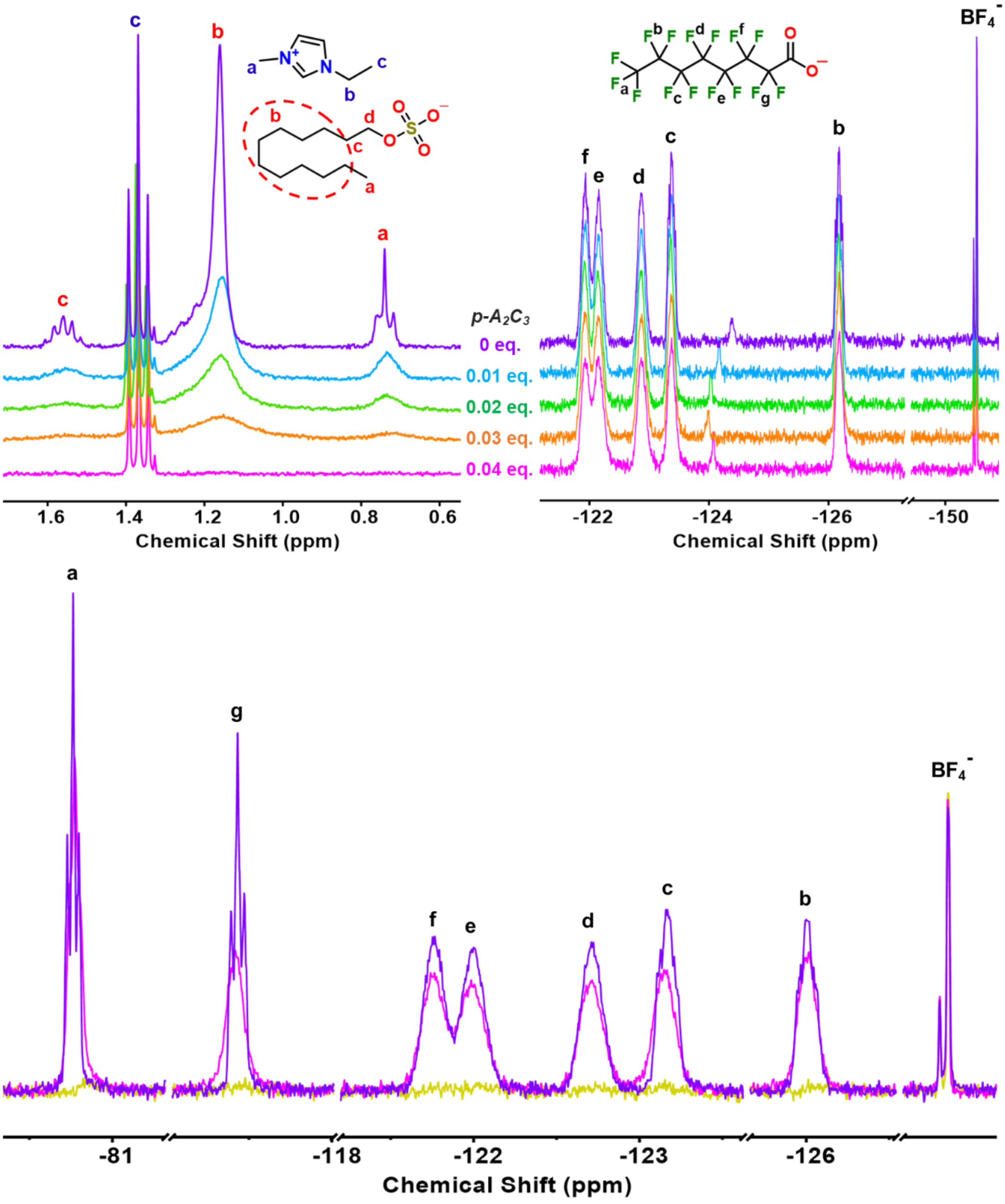

Figure S11. *^{1}H-NMR (300 MHz,298 K) of SDS (1 mM) and reference (3 mM). Zoom in the surfactant's signals after the addition of up to 0.04 equivalents of* ***p-cage****, showing signal decay until complete removal (left) and ^{19}F-NMR (282 MHz, D_2O) of PFOA (1 mM) and $NaBF_4$ (1 mM) from the same NMR tube, showing no signal decay after the addition of 0.04 equivalents of* ***p-cage****. Below, zoom in the PFOA signals, after the addition of 0 (purple), 0.04 (pink) and 0.1 equivalents of* ***p-cage****, showing complete removal.*

4.2. Caprylate – PFOA mixture

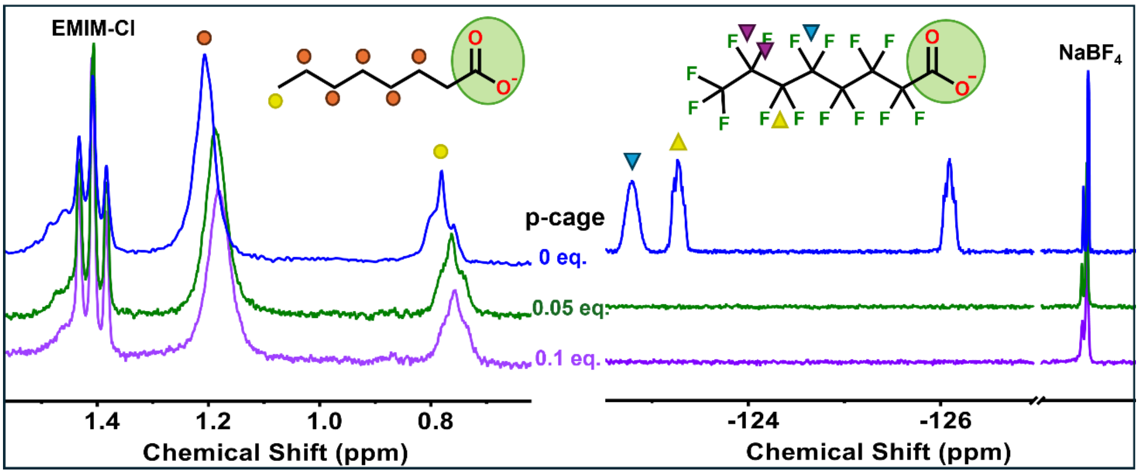

Figure S12. *^{1}H-NMR (300 MHz,298 K) of caprylate (1 mM) and reference (1 mM) and ^{19}F-NMR (282 MHz, D_2O) of PFOA (1 mM) and $NaBF_4$ (1 mM), both from the same NMR tube. Zoom in the surfactant's signals after the addition of up to 0.1 equivalents of* ***p-cage*** *(purple), showing no decay for caprylate while PFOA was completely removed after the addition of 0.05 equivalents of* ***p-cage*** *(green).*

4.3. PFHxA – PFOA mixture

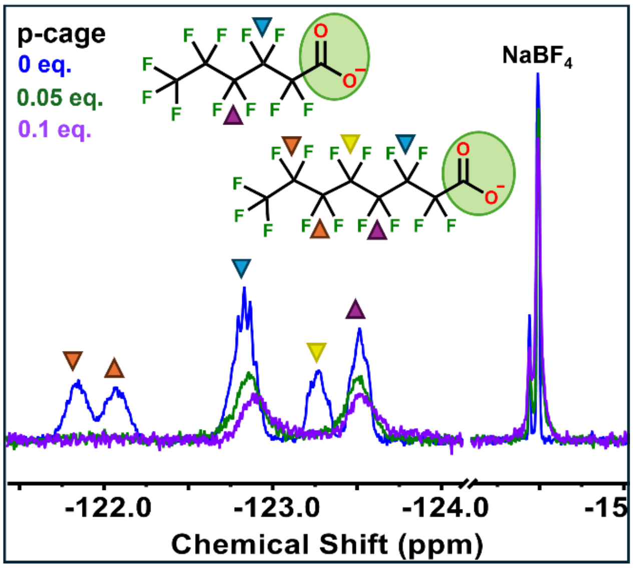

Figure S13. *^{19}F-NMR (282 MHz, D_2O) of PFOA (1 mM), PFHxA (1 mM) and $NaBF_4$ (1 mM), both from the same NMR tube. Zoom in the surfactant's signals after the addition of up to 0.1 equivalents of* ***p-cage*** *(purple), showing almost no decay for PFHxA, while PFOA was completely removed after the addition of 0.05 equivalents of* ***p-cage*** *(green).*

5. Titration of three-component mixture

5.1. PFOA – SDS – SHS mixture

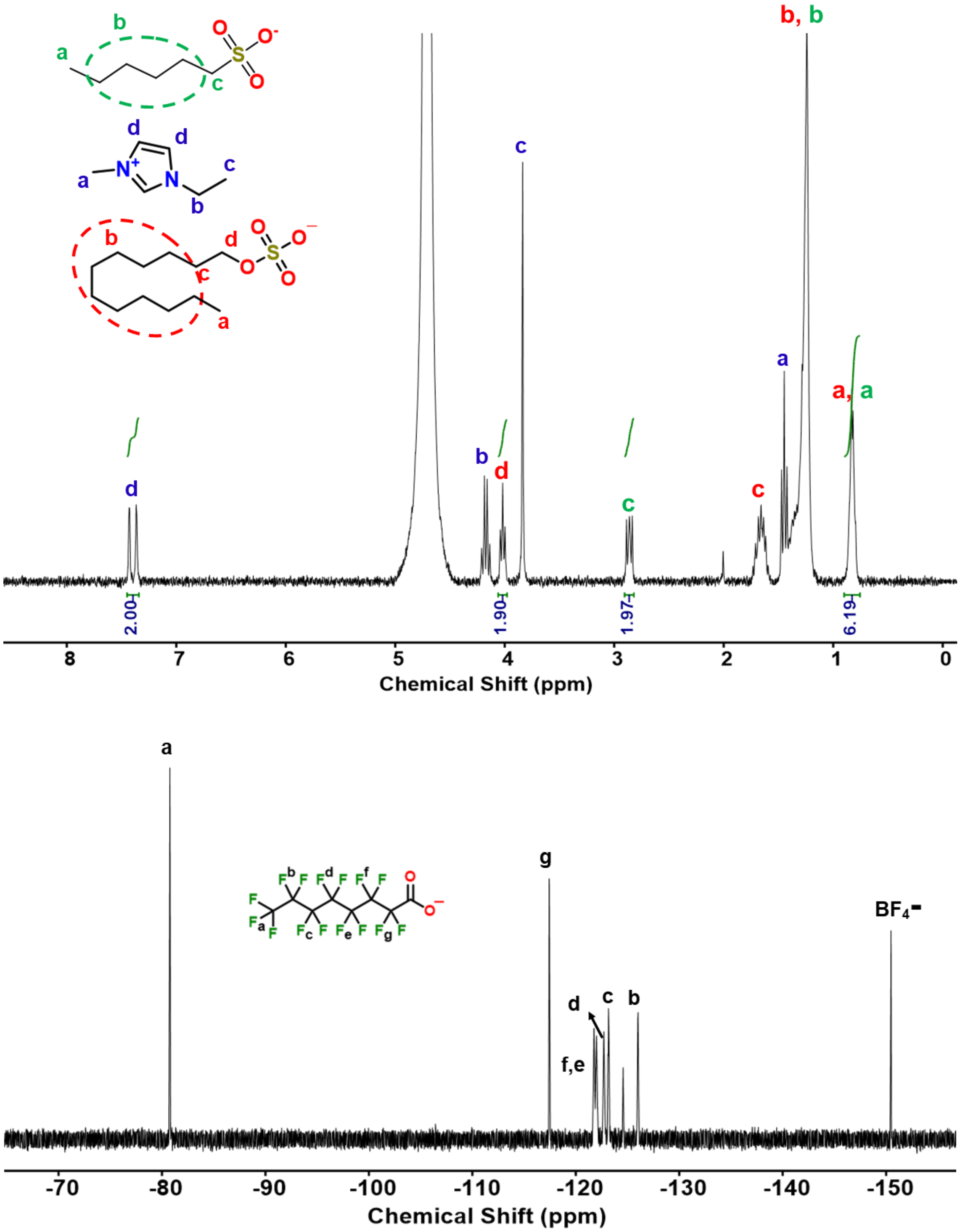

Figure S14. *Tube containing SDS (1 mM), SHS (1 mM), 1-ethyl-3-methylimidazolium chloride (1 mM), PFOA (1mM) and $NaBF_4$ (0.5 mM) in D_2O, above: ^{1}H-NMR (300 MHz,298 K); below: ^{19}F-NMR (282 MHz, 298 K).*

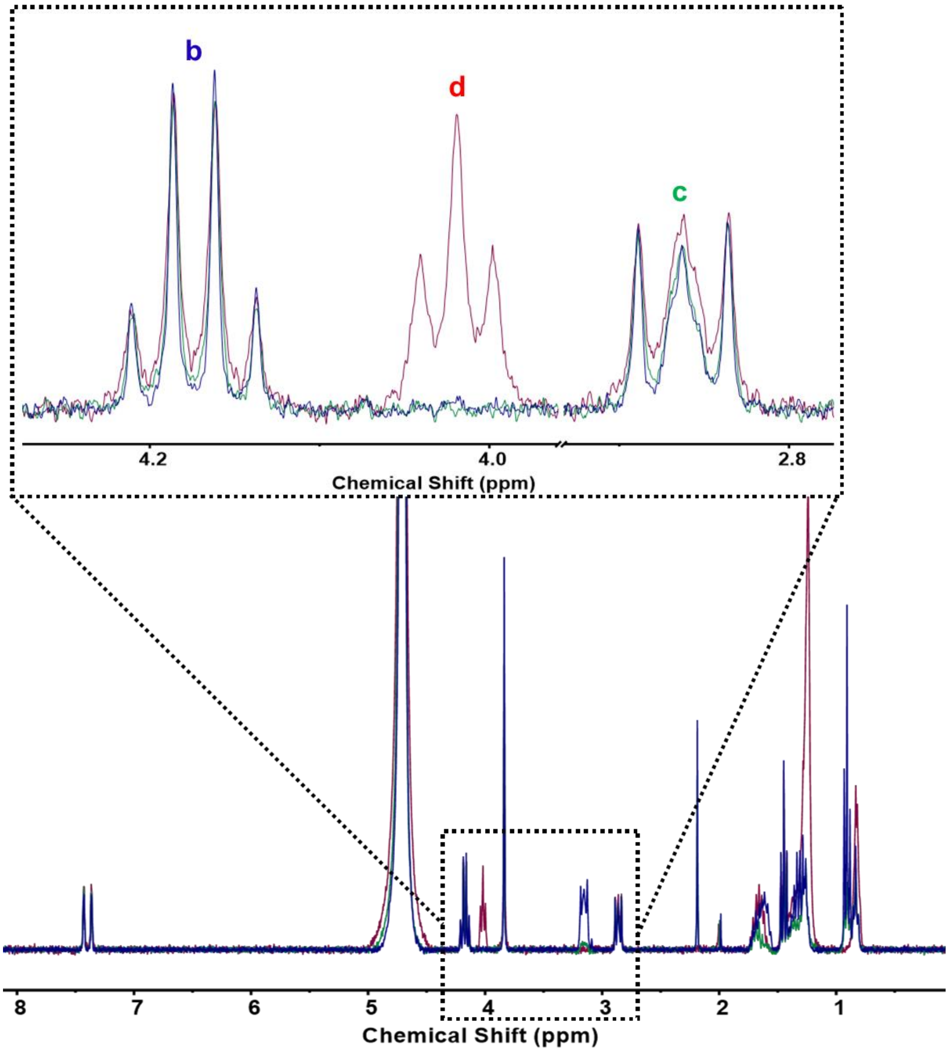

Figure S15. *Zoom in the ^{1}H-NMR surfactant's signals (from Figure S13 NMR) after the addition of 0.04 (green) and 0.1 equivalents (blue) of* ***p-cage****, showing complete removal of SDS after the addition of 0.04 equivalents, whereas SHS remains in solution, even after the addition of 0.1 equivalents.*

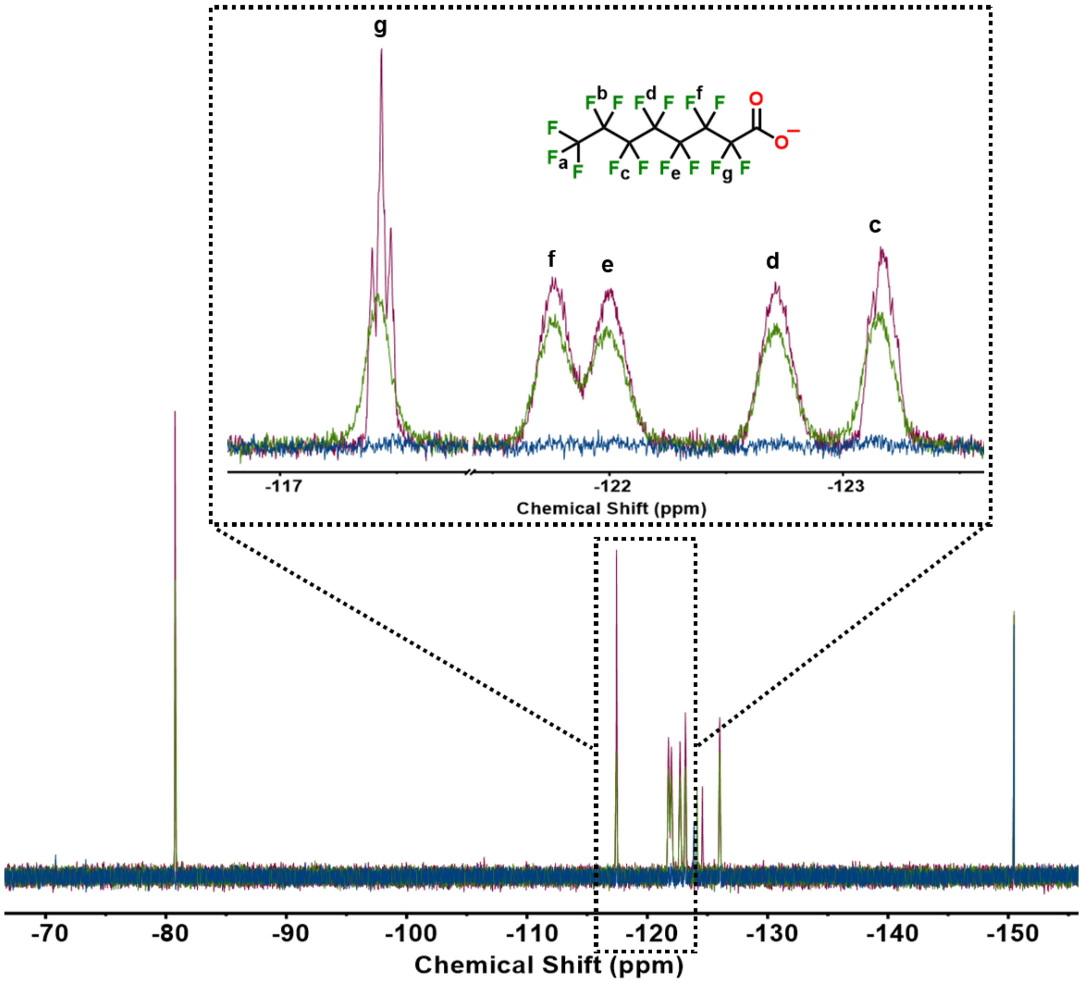

Figure S16. *Zoom in the ^{19}F-NMR PFOA signals from Figure S13 NMR after the addition of 0.04 (green) and 0.1 equivalents (blue) of* ***p-cage****, showing complete removal of PFOA after the addition of 0.1 equivalents.*
